# Preoperative stenting in insulinoma located nearby pancreatic duct: a case report and literature review

**DOI:** 10.1093/jscr/rjab121

**Published:** 2021-04-29

**Authors:** Miroslav Pindura, Lenka Nosáková, Roman Kyčina, Martin Vojtko, Peter Bánovčin, Michal Demeter, L'udovit Laca

**Affiliations:** Surgery Department and Transplant Center, JFM CU, Jessenius Faculty of Medicine in Martin (JFM CU), Comenius University, Bratislava, Slovakia; Clinic of Internal Medicine—Gastroenterology, JFM CU, Jessenius Faculty of Medicine in Martin (JFM CU), Comenius University, Bratislava, Slovakia; Surgery Department and Transplant Center, JFM CU, Jessenius Faculty of Medicine in Martin (JFM CU), Comenius University, Bratislava, Slovakia; Surgery Department and Transplant Center, JFM CU, Jessenius Faculty of Medicine in Martin (JFM CU), Comenius University, Bratislava, Slovakia; Clinic of Internal Medicine—Gastroenterology, JFM CU, Jessenius Faculty of Medicine in Martin (JFM CU), Comenius University, Bratislava, Slovakia; Clinic of Internal Medicine—Gastroenterology, JFM CU, Jessenius Faculty of Medicine in Martin (JFM CU), Comenius University, Bratislava, Slovakia; Surgery Department and Transplant Center, JFM CU, Jessenius Faculty of Medicine in Martin (JFM CU), Comenius University, Bratislava, Slovakia

## Abstract

Insulinoma is a rare functional neuroendocrine tumor of pancreas. The only recommended treatment is surgical removal. We present a case of a 46-year-old female patient who underwent the enucleation of insulinoma localized nearby pancreatic main duct after preoperative endoscopic insertion of pancreatic stent. The tumor was safely identified during the surgery and was enucleated without injury of pancreatic duct or postoperative complications.

## INTRODUCTION

Insulinomas are functional neuroendocrine tumors with sporadic incidence. Almost every patient suffers from spontaneous hypoglycemia resulting from tumor autonomic insulin overproduction. Insulinomas are almost always localized in pancreas [1]. Usually, they are small and preoperative localization is difficult. The only recommended treatment is surgical removal. Due to ENETS guidelines, standard surgical approach should include exploration of pancreas with IOUS and palpation [1, 2]. Some patients can undergo the laparoscopic resection [3, 4]. Tumor enucleation should be the procedure of choice for selected patients. In case of close anatomical proximity to the pancreatic duct, distal pancreatectomy or proximal pancreaticoduodenectomy may also be required [1, 2]. In this case report, we present a surgical treatment of insulinoma of pancreatic head localized nearby pancreatic duct with preoperative stenting of pancreatic duct.

## CLINICAL CASE REPORT

**Figure 1:**
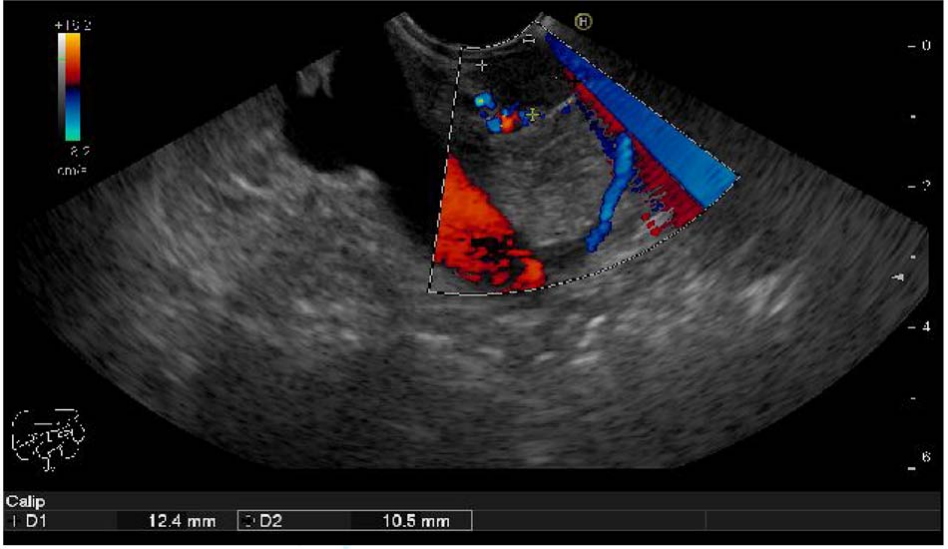
Hypoechogenic round shape tumor in the head of pancreas (between two marks)–EUS.

**Figure 2:**
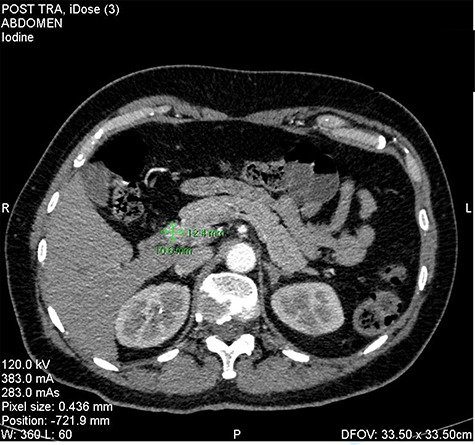
Insulinoma in the head of the pancreas—CT picture in arterial phase (as shown green marks).

**Figure 3:**
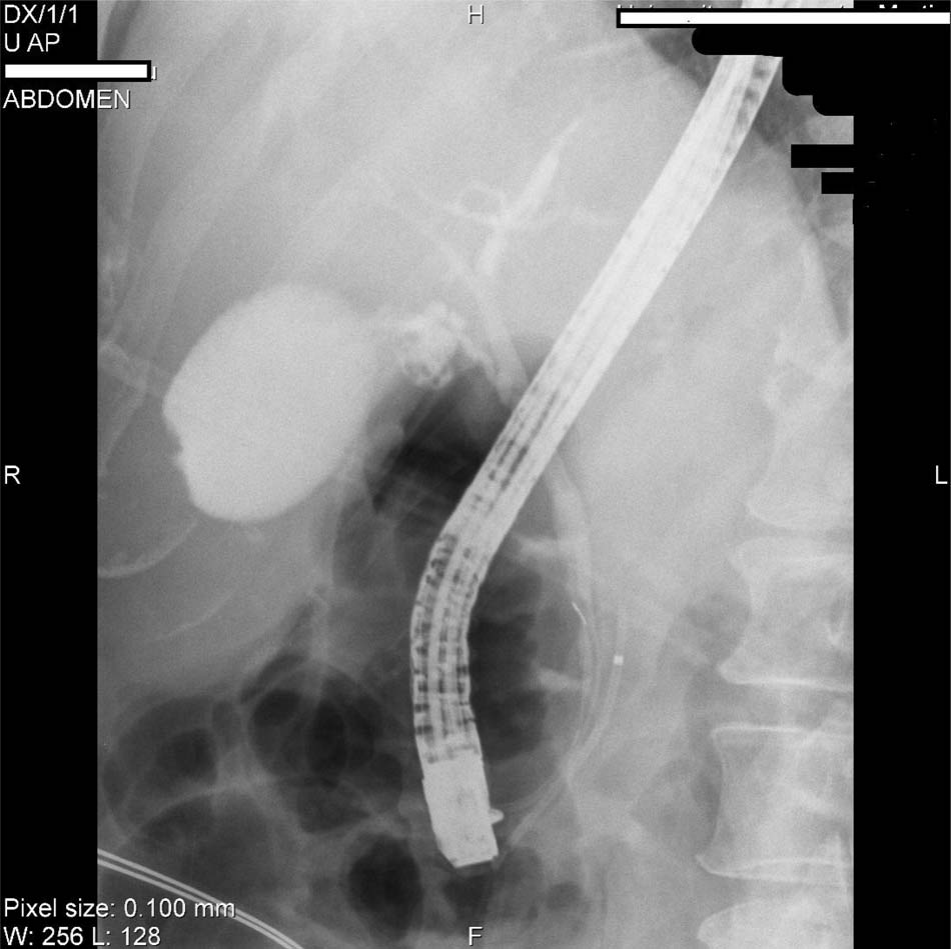
ERCP picture of inserted stents in pancreatic (near the arrow) and bile duct.

**Figure 4:**
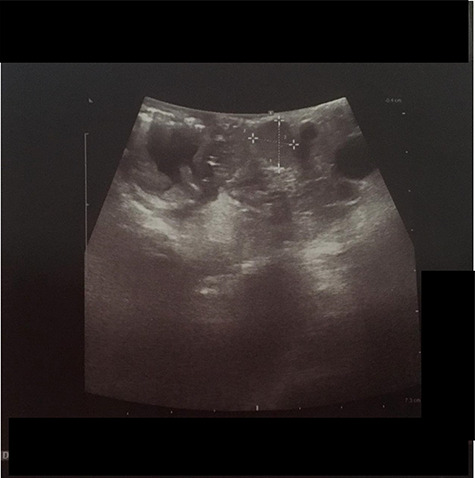
IOUS (intraoperative ultrasound) performed during surgery shown hypoechogenic lesion in the head of pancreas (between white marks).

**Figure 5:**
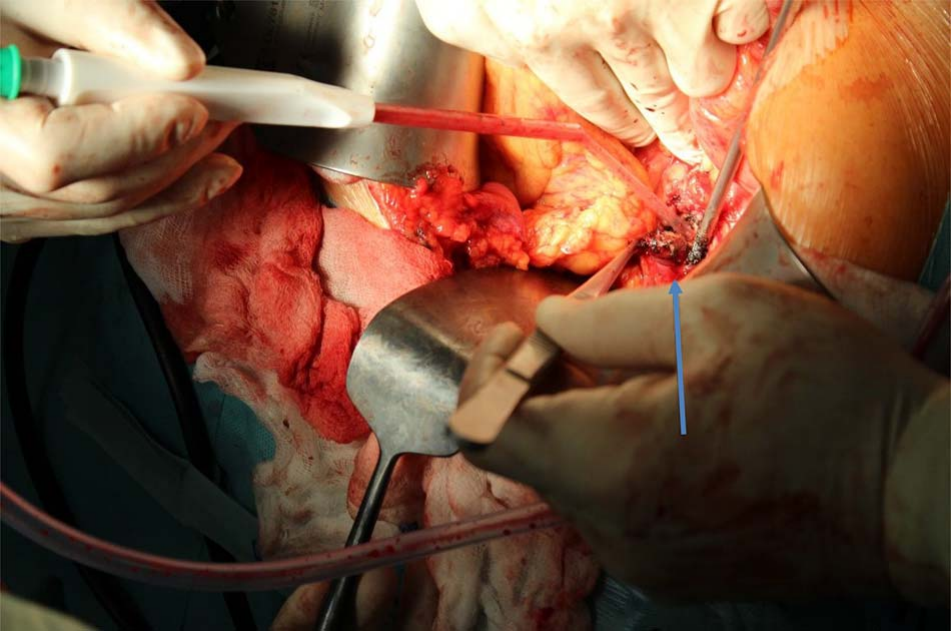
Enucleation of insulinoma by using of harmonic skalpel (arrow shows enucleated insulinoma).

**Figure 6:**
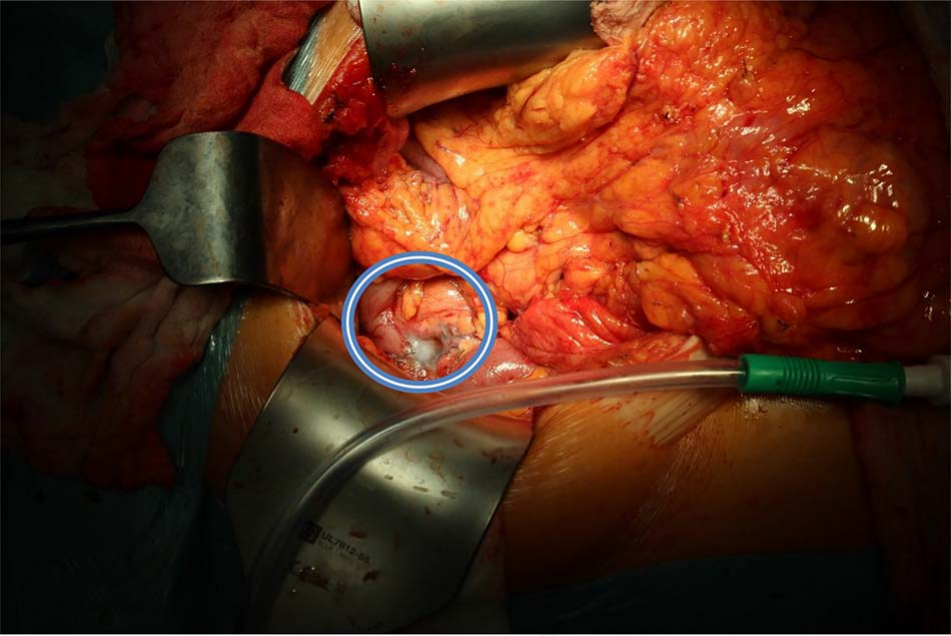
Pancreatic defect after enucleation of insulinoma filled with tissue glue (in the circle).

**Figure 7:**
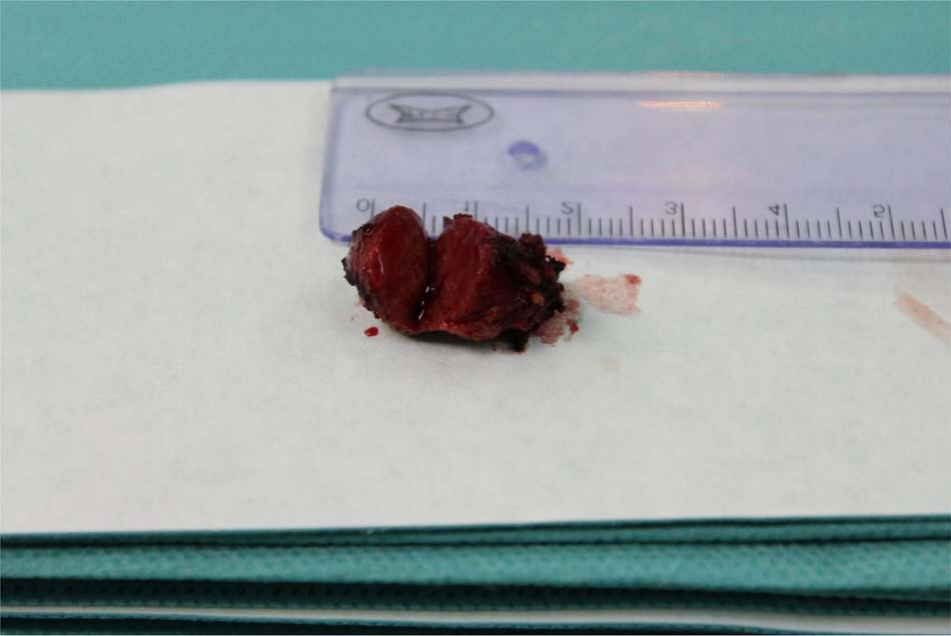
Enucleated insulinoma cross sectional image.

We present the case of 46-year-old female patient suffering from spontaneous hypoglycemia. She was admitted to our hospital due to worsening clinical symptoms. At first, she underwent a 72-hour monitored fast and biochemical testing for endogenous hyperinsulinism, which proved positive. Once the other causes of spontaneous hypoglycemia were excluded, we started the process of localization of insulinoma. Then the patient underwent endoscopic ultrasonography (EUS) of pancreas with the find of hypoechogenic, well demarcated, hypervascular tumor in the head of pancreas size of 12.4 × 10.5 mm (Fig.1). Computed tomography (CT) examination of abdomen followed up and tumor of pancreatic head, corresponding with the diagnosis of insulinoma, was found (Fig. 2). It was located nearby pancreatic and biliary duct. Because of serious clinical symptoms, an early surgical treatment was indicated. Based on the evaluation of the surgeon to perform the less radical surgery, it was suggested to enucleate the tumor with preoperative stenting of pancreatic and biliary duct. Two days before the surgery, ERCP with insertion of pancreatic and duodenobiliary stent was performed (Fig. 3). With regard to the patient's preference and overweight, laparotomic approach was selected. Determination of the exact position of tumor was difficult, insulinoma was hardly palpable. We used intraoperative ultrasonography to identify the exact location of tumor (Fig. 4). Then the surgeon palpated the inserted stents and the insulinoma was identified intrapancreatically, 3–4 mm nearby pancreatic and bile duct. Enucleation of tumor was performed by harmonic scalpel (Fig. 5). During the surgery, the right position of stents was verified by touch. After enucleation, there was no presence of bleeding, the defect was filled with tissue glue (Fig. 6). Macroscopically, tumor was round shaped, well capsulated, size 1 cm (Fig. 7). During postsurgery period, the patient was stable and with no signs of complication. On the sixth postoperative day, endoscopic removal of pancreatic stent was carried out. Patient was discharged from the hospital on the eight postoperative day. Duodenobiliary stent was left in *ductus choledochus* for 1 month.

## DISCUSSION

We reported our experience with the surgical treatment in patient with insulinoma to the preoperative management. Insulinomas are rare, but most common functional pancreatic neuroendocrine tumors [1, 5, 6]. The ability to localize the tumor before surgery is crucial in the management of these lesions. Standard diagnostic modalities such as ultrasonography, CT, magnetic resonance imaging have low sensitivity and allow to localize tumor only in 10–40% of cases [1]. However, the use of specific imaging methods such as SRS (somatostatin receptor scintigraphy), positron emission tomography/CT labeled with 68-Ga-DATOTOC, has its limits in case of insulinoma due to the low production of somatostatin receptors [1]. The most sensitive examination in localization of tumor is EUS. Besides that, it can predict possibilities of the enucleation and specify the proximity to pancreatic and bile duct.

Approach to treatment of insulinoma has some characteristics. Surgery is still the only possible curative treatment. According to ENETS consensus and guideline in management of functional pancreatic neuroendocrine tumors, standard surgical treatment should include pancreas exploration both palpation and IOUS [1]. From surgical point of view, both biochemical confirmation of tumor and localization are necessary. If tumor is localized more than 2–3 mm away from pancreatic duct, the enucleation should be performed. The enucleation is indicated in case of single, superficial, well-demarcated tumor, <2 cm with favorable localization to pancreatic duct [7]. It is relatively safe procedure with low mortality rate; however, the morbidity is similar to classic pancreatic resection with common development of pancreatic fistulas. The occurrence of postoperative morbidity is relatively high and varies from 15% to 77% according to the recent study [8–11]. The occurrence of pancreatic fistula may decrease with construction of pancreaticoenteroanastomosis (Roux-en-Y) located in the pancreatic defect [12, 13]. The most common occurring complication is development of pancreatic fistula. If enucleation is not possible, partial pancreatic resection is performed. If we are not able to localize insulinoma preoperatively, surgical exploration is indicated. Blind resection of a part of pancreas is not recommended.

The topic of preoperative stenting of pancreatic duct (EPS) in a case of pancreatic insulinoma as prevention to development of pancreatic fistula is not researched well enough. There are only a few case reports in literature describing and using this method [14]. In pancreatic surgery, there is an attempt to prevent the formation of a fistula. In recent year, various procedures have been used, such as using of harmonic scalpel, automatic stapler, fibrin glue sealing or octreotide administration [14, 15]. The use of preoperative stenting is well known in case of pancreatic cancer with biliary obstruction, but there are some studies and works using preoperative stenting before distal pancreatectomy [14, 15]. Advantage of stent placement against enucleation is avoiding the risk of pancreatic duct injury during surgery and postoperative pancreatic fistula development [14]. Moreover, indwelling EPS reduces lifting of the pancreatic duct during tumor enucleation [14]. There is a risk connected with this procedure, such as development of pancreatitis. There is a need of randomized study focusing on effectiveness and complications of this approach.

## CONCLUSION

Multidisciplinary assessment in management of insulinoma is still the best option. Decision to make surgery should be based on consensus surgeon—gastroenterologist—patient. Preoperational stenting as prevention of postoperative pancreatic fistula can be considered in localization of insulinoma nearby the pancreatic duct.
